# 2-Hy­droxy­methyl-1,3-dimethyl-1*H*-imidazol-3-ium triiodide

**DOI:** 10.1107/S1600536813020266

**Published:** 2013-07-27

**Authors:** Mohamed El Hadi Said, Abdelmalek Bouraiou, Sofiane Bouacida, Hocine Merazig, Ali Belfaitah, Aissa Chibani

**Affiliations:** a Unité de Recherche de Chimie de l’Environnement et Moléculaire Structurale, CHEMS, Université Constantine1, 25000 , Algeria; b Laboratoire des Produits Naturels d’Origine Végétale et de Synthèse, Organique, PHYSYNOR, Université Constantine1, 25000 Constantine, Algeria

## Abstract

The crystal packing of the title salt, C_6_H_11_N_2_O^+^·I_3_^−^, can be described as consisting of alternating layers of cations and anions parallel to the (100) plane along the *a-*axis direction. The components are linked by O—H⋯I, C—H⋯I and C—H⋯O interactions, generating a three-dimensional network. The O atom deviates from the imidazol ring by 0.896 (2) Å.

## Related literature

For the importance of heterocyclic compounds and their applications, see: Pandey *et al.* (2009 ▶); Nasser (2000 ▶). For the biological activity of imidazole and imidazolium derivatives, see: Ucucu *et al.* (2001 ▶); Dominianni *et al.* (1989 ▶); Ozkay *et al.* (2010 ▶). For our previous work on imidazole derivatives, see: Bahnous *et al.* (2012 ▶); Zama *et al.* (2013 ▶); Chelghoum *et al.* (2011 ▶).
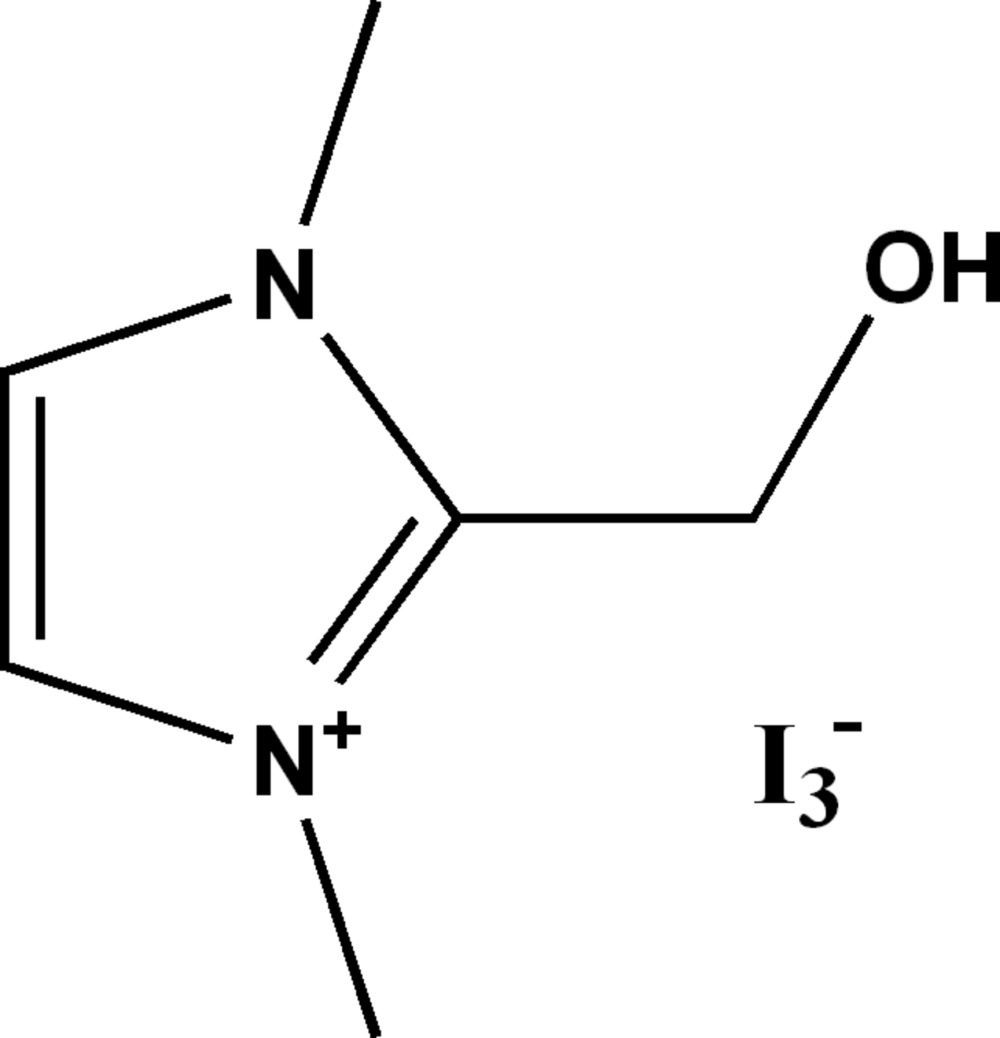


## Experimental

### 

#### Crystal data

C_6_H_11_N_2_O^+^·I_3_^−^
*M* *_r_* = 507.87
Monoclinic, 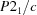

*a* = 7.1647 (8) Å
*b* = 15.5586 (19) Å
*c* = 11.3201 (13) Å
β = 96.026 (7)°
*V* = 1254.9 (3) Å^3^
*Z* = 4
Mo *K*α radiation
μ = 7.44 mm^−1^
*T* = 150 K
0.24 × 0.03 × 0.02 mm

#### Data collection

Bruker APEXII diffractometer
Absorption correction: multi-scan (*SADABS*; Sheldrick, 2002 ▶) *T*_min_ = 0.781, *T*_max_ = 1.000
7061 measured reflections
2222 independent reflections
2104 reflections with *I* > 2σ(*I*)
*R*_int_ = 0.022

#### Refinement

*R*[*F* ^2^ > 2σ(*F* ^2^)] = 0.015
*wR*(*F* ^2^) = 0.034
*S* = 1.15
2222 reflections
112 parameters
H-atom parameters constrained
Δρ_max_ = 0.45 e Å^−3^
Δρ_min_ = −0.47 e Å^−3^

### 

Data collection: *APEX2* (Bruker, 2006 ▶); cell refinement: *SMART* (Bruker, 2006 ▶); data reduction: *SMART*; program(s) used to solve structure: *SIR2002* (Burla *et al.*, 2005 ▶); program(s) used to refine structure: *SHELXL97* (Sheldrick, 2008 ▶); molecular graphics: *ORTEP-3 for Windows* (Farrugia, 2012 ▶) and *DIAMOND* (Brandenburg & Berndt, 2001 ▶); software used to prepare material for publication: *WinGX* (Farrugia, 2012) ▶ and *CRYSCAL* (T. Roisnel, local program).

## Supplementary Material

Crystal structure: contains datablock(s) I. DOI: 10.1107/S1600536813020266/hg5333sup1.cif

Structure factors: contains datablock(s) I. DOI: 10.1107/S1600536813020266/hg5333Isup2.hkl

Supplementary material file. DOI: 10.1107/S1600536813020266/hg5333Isup3.cml

Additional supplementary materials:  crystallographic information; 3D view; checkCIF report

## Figures and Tables

**Table 1:** Hydrogen-bond geometry (Å, °)

*D*—H⋯*A*	*D*—H	H⋯*A*	*D*⋯*A*	*D*—H⋯*A*
O1—H1⋯I1^i^	0.82	3.03	3.741 (2)	146
C1—H1*B*⋯I3^ii^	0.97	3.05	3.924 (3)	151
C4—H4⋯O1^iii^	0.93	2.60	3.421 (4)	148

Symmetry codes: (i) 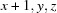; (ii) 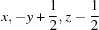; (iii) 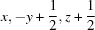.

**Fig. 1.**
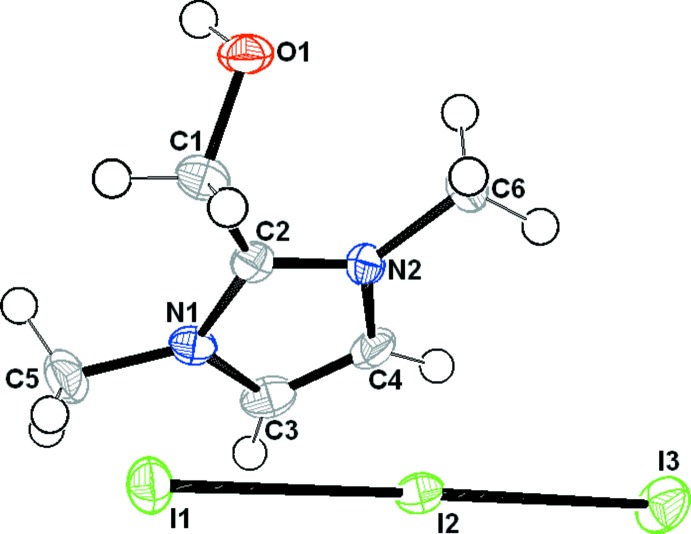
The molecular geometry of (I) with the atom-labelling scheme. Displacement ellipsoids are drawn at the 50% probability level. H atoms are represented as small spheres of arbitrary radius.

**Fig. 2.**
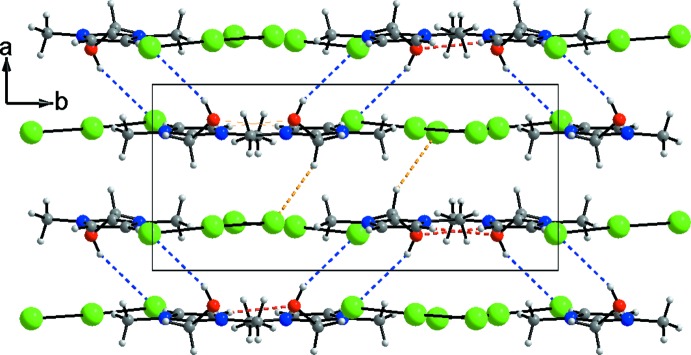
Alternating layers of (I) viewed down the c axis showing hydrgen bond as dashed line.

## supplementary crystallographic information

### Comment

Heterocyclic compounds have so far been synthesized mainly due to the wide range of biological activities. At present, the role of heterocyclic compounds has become increasingly important in designing new class of structural entities of medicinal importance (Pandey *et al.*, 2009; Nasser, 2000). Imidazole is a nitrogen containing heterocyclic ring which possesses biological and pharmaceutical importance (Ozkay, *et al.*, 2010). It forms the main structure of some well known components of human organisms, *i.e.* the amino acid histidine, Vit-B12, a component of DNA base structure and purines, histamine and biotin (Ucucu, *et al.*, 2001). In other hand, imidazolium salts are known for the wide range of their biological activity. A large variety of these salts have been used as anti-inflammatory, antibacterial, antifungal and thromboxane synthetase inhibitior (Dominianni *et al.*, 1989). In continuation of our studies on imidazole derivatives (Bahnous *et al.*, 2012; Zama *et al.*, 2013 and Chelghoum *et al.*, 2011). We report herein the synthesis and crystal structure of a new imidazolium salt, I, bearing two methyl groups at C-1 and C-3 positions, a hydroxymethyl at C-2 and a triiodide anion that balance the charge.

The molecular geometry and the atom-numbering scheme of (I) are shown in Fig. 1. The asymmetric unit of title molecule, C_6_H_11_N_2_O, I_3_, contains a 1,3-dimethyl-2-hydroxymethylidazolium cation and triiodide anion. The crystal packing can be described as alternating layers parallel to the (100) plane along the *a* axis, where triiodide anion is located in these layers (Fig. 2) and they are linked together by O—H···I, C—H···I and C—H···O intermolecular hydrogen bonds As shown in the Figure 2, the imidazol rings of the symmetry related layers are intercalated, however the centroid to centroid distance between the imidazol rings are too long (5.3400 (19) and 5.6641 (19) Å) for considering π-π interactions. These interaction bonds link the molecules within the layers and also link the layers together, reinforcing the cohesion of the ionic structure. Hydrogen-bonding parameters are listed in table 1.

### Experimental

The treatment of 1,3-dimethyl-2-hydroxymethylimidazolium iodide (Chelghoum *et al.*, 2011) with diluted sulfuric acid solution, during ten days in opened flask for slow evaporation, gave the title compound as a brown crystals. The crystals are filtered off and washed with water. Suitable crystal of compound (I) was selected and X-ray crystallographic analysis confirmed the structural assignment (Fig. 1).

### Refinement

Approximate positions for all the H atoms were first obtained from the difference electron density map. However, the H atoms were situated into idealized positions and the H-atoms have been refined within the riding atom approximation. The applied constraints were as follow: C_aryl_—H_aryl_ = 0.93 Å; C_methylene_—H_methylene_ = 0.97 Å; C_methyl_—H_methyl_ = 0.96 Å and C_hydroxy_—H_hydroxy_ = 0.82 Å; The idealized methyl group was allowed to rotate about the C—C bond during the refinement by application of the command AFIX 137 in *SHELXL97* (Sheldrick, 2008). *U*_iso_(H_methyl_ or _hydroxy_) = 1.5*U*_eq_(C_methyl_ or _hydroxy_) or *U*_iso_(H_aryl_ or H_methylene_) = 1.2 *U*_eq_(C_aryl_ or C_methylene_).

### Crystal data

**Table:**

C_6_H_11_N_2_O^+^·I_3_^−^	*F*(000) = 912
*M**_r_* = 507.87	*D*_x_ = 2.688 Mg m^−^^3^
Monoclinic, *P*2_1_/*c*	Mo *K*α radiation, λ = 0.71073 Å
Hall symbol: -P 2ybc	Cell parameters from 5058 reflections
*a* = 7.1647 (8) Å	θ = 2.2–25.1°
*b* = 15.5586 (19) Å	µ = 7.44 mm^−^^1^
*c* = 11.3201 (13) Å	*T* = 150 K
β = 96.026 (7)°	Stick, brown
*V* = 1254.9 (3) Å^3^	0.24 × 0.03 × 0.02 mm
*Z* = 4	

### Data collection

**Table:**

Bruker APEXII diffractometer	2104 reflections with *I* > 2σ(*I*)
Graphite monochromator	*R*_int_ = 0.022
CCD rotation images, thin slices scans	θ_max_ = 25.1°, θ_min_ = 2.6°
Absorption correction: multi-scan (*SADABS*; Sheldrick, 2002)	*h* = −8→8
*T*_min_ = 0.781, *T*_max_ = 1.000	*k* = −18→18
7061 measured reflections	*l* = −13→13
2222 independent reflections	

### Refinement

**Table:**

Refinement on *F*^2^	Primary atom site location: structure-invariant direct methods
Least-squares matrix: full	Secondary atom site location: difference Fourier map
*R*[*F*^2^ > 2σ(*F*^2^)] = 0.015	Hydrogen site location: inferred from neighbouring sites
*wR*(*F*^2^) = 0.034	H-atom parameters constrained
*S* = 1.15	*w* = 1/[σ^2^(*F*_o_^2^) + 1.5935*P*] where *P* = (*F*_o_^2^ + 2*F*_c_^2^)/3
2222 reflections	(Δ/σ)_max_ = 0.001
112 parameters	Δρ_max_ = 0.45 e Å^−^^3^
0 restraints	Δρ_min_ = −0.47 e Å^−^^3^

### Special details

**Table:**

Geometry. All e.s.d.'s (except the e.s.d. in the dihedral angle between two l.s. planes) are estimated using the full covariance matrix. The cell e.s.d.'s are taken into account individually in the estimation of e.s.d.'s in distances, angles and torsion angles; correlations between e.s.d.'s in cell parameters are only used when they are defined by crystal symmetry. An approximate (isotropic) treatment of cell e.s.d.'s is used for estimating e.s.d.'s involving l.s. planes.
Refinement. Refinement of *F*^2^ against ALL reflections. The weighted *R*-factor *wR* and goodness of fit *S* are based on *F*^2^, conventional *R*-factors *R* are based on *F*, with *F* set to zero for negative *F*^2^. The threshold expression of *F*^2^ > σ(*F*^2^) is used only for calculating *R*-factors(gt) *etc*. and is not relevant to the choice of reflections for refinement. *R*-factors based on *F*^2^ are statistically about twice as large as those based on *F*, and *R*- factors based on ALL data will be even larger.

### Fractional atomic coordinates and isotropic or equivalent isotropic displacement parameters (Å^2^)

**Table:**

	*x*	*y*	*z*	*U*_iso_*/*U*_eq_	
I2	0.23583 (2)	0.148350 (12)	0.326494 (15)	0.01269 (6)	
I1	0.19816 (3)	−0.006510 (12)	0.171029 (17)	0.01684 (6)	
I3	0.25836 (3)	0.297933 (14)	0.474053 (18)	0.02126 (6)	
N2	0.7383 (3)	0.17050 (16)	0.3419 (2)	0.0144 (5)	
O1	0.8099 (3)	0.15052 (14)	0.09130 (18)	0.0177 (5)	
H1	0.9119	0.1286	0.0833	0.027*	
N1	0.7438 (3)	0.03209 (16)	0.3568 (2)	0.0141 (5)	
C2	0.7275 (4)	0.09763 (19)	0.2795 (3)	0.0132 (6)	
C5	0.7344 (4)	−0.0598 (2)	0.3266 (3)	0.0209 (7)	
H5A	0.61	−0.0738	0.292	0.031*	
H5B	0.7643	−0.0933	0.3972	0.031*	
H5C	0.8227	−0.0723	0.2707	0.031*	
C6	0.7239 (4)	0.2584 (2)	0.2943 (3)	0.0196 (7)	
H6A	0.8421	0.2751	0.2686	0.029*	
H6B	0.6918	0.2971	0.3551	0.029*	
H6C	0.6285	0.2605	0.2282	0.029*	
C3	0.7663 (4)	0.0651 (2)	0.4703 (3)	0.0195 (7)	
H3	0.7811	0.0336	0.5405	0.023*	
C4	0.7630 (4)	0.1512 (2)	0.4610 (3)	0.0171 (7)	
H4	0.7751	0.1904	0.5234	0.021*	
C1	0.6972 (4)	0.0911 (2)	0.1468 (3)	0.0160 (6)	
H1A	0.7269	0.0332	0.1228	0.019*	
H1B	0.566	0.1018	0.1204	0.019*	

### Atomic displacement parameters (Å^2^)

**Table:**

	*U*^11^	*U*^22^	*U*^33^	*U*^12^	*U*^13^	*U*^23^
I2	0.01199 (10)	0.01601 (11)	0.01018 (10)	−0.00047 (7)	0.00176 (7)	0.00073 (7)
I1	0.02125 (11)	0.01258 (11)	0.01685 (11)	−0.00009 (8)	0.00283 (8)	−0.00207 (8)
I3	0.02175 (11)	0.02351 (12)	0.01884 (12)	−0.00312 (8)	0.00360 (8)	−0.00943 (8)
N2	0.0138 (12)	0.0121 (13)	0.0176 (14)	0.0021 (10)	0.0039 (10)	0.0011 (11)
O1	0.0196 (10)	0.0200 (12)	0.0144 (11)	−0.0001 (9)	0.0058 (8)	0.0043 (9)
N1	0.0139 (12)	0.0159 (13)	0.0122 (12)	−0.0006 (10)	0.0008 (9)	0.0034 (11)
C2	0.0075 (13)	0.0149 (15)	0.0172 (15)	0.0021 (11)	0.0017 (11)	0.0017 (12)
C5	0.0276 (17)	0.0114 (16)	0.0234 (17)	0.0011 (13)	0.0022 (13)	0.0032 (13)
C6	0.0235 (16)	0.0124 (16)	0.0239 (17)	0.0016 (13)	0.0075 (13)	0.0029 (13)
C3	0.0193 (15)	0.0275 (19)	0.0117 (15)	0.0002 (13)	0.0021 (12)	0.0025 (13)
C4	0.0182 (15)	0.0213 (17)	0.0127 (15)	−0.0006 (13)	0.0054 (12)	−0.0039 (13)
C1	0.0158 (14)	0.0176 (16)	0.0141 (15)	−0.0007 (12)	−0.0004 (11)	0.0042 (13)

### Geometric parameters (Å, º)

**Table:**

I2—I3	2.8594 (4)	C5—H5A	0.96
I2—I1	2.9792 (4)	C5—H5B	0.96
N2—C2	1.334 (4)	C5—H5C	0.96
N2—C4	1.374 (4)	C6—H6A	0.96
N2—C6	1.470 (4)	C6—H6B	0.96
O1—C1	1.417 (3)	C6—H6C	0.96
O1—H1	0.82	C3—C4	1.344 (5)
N1—C2	1.341 (4)	C3—H3	0.93
N1—C3	1.377 (4)	C4—H4	0.93
N1—C5	1.470 (4)	C1—H1A	0.97
C2—C1	1.499 (4)	C1—H1B	0.97
			
I3—I2—I1	178.026 (8)	N2—C6—H6B	109.5
C2—N2—C4	109.2 (3)	H6A—C6—H6B	109.5
C2—N2—C6	126.8 (3)	N2—C6—H6C	109.5
C4—N2—C6	124.0 (3)	H6A—C6—H6C	109.5
C1—O1—H1	109.5	H6B—C6—H6C	109.5
C2—N1—C3	108.6 (3)	C4—C3—N1	107.4 (3)
C2—N1—C5	126.1 (3)	C4—C3—H3	126.3
C3—N1—C5	125.3 (3)	N1—C3—H3	126.3
N2—C2—N1	107.7 (3)	C3—C4—N2	107.1 (3)
N2—C2—C1	125.7 (3)	C3—C4—H4	126.4
N1—C2—C1	126.6 (3)	N2—C4—H4	126.4
N1—C5—H5A	109.5	O1—C1—C2	111.7 (2)
N1—C5—H5B	109.5	O1—C1—H1A	109.3
H5A—C5—H5B	109.5	C2—C1—H1A	109.3
N1—C5—H5C	109.5	O1—C1—H1B	109.3
H5A—C5—H5C	109.5	C2—C1—H1B	109.3
H5B—C5—H5C	109.5	H1A—C1—H1B	107.9
N2—C6—H6A	109.5		
			
C4—N2—C2—N1	0.4 (3)	C2—N1—C3—C4	0.2 (3)
C6—N2—C2—N1	−178.8 (2)	C5—N1—C3—C4	−178.4 (3)
C4—N2—C2—C1	178.8 (3)	N1—C3—C4—N2	0.1 (3)
C6—N2—C2—C1	−0.4 (4)	C2—N2—C4—C3	−0.3 (3)
C3—N1—C2—N2	−0.4 (3)	C6—N2—C4—C3	179.0 (3)
C5—N1—C2—N2	178.2 (2)	N2—C2—C1—O1	44.3 (4)
C3—N1—C2—C1	−178.7 (3)	N1—C2—C1—O1	−137.6 (3)
C5—N1—C2—C1	−0.2 (4)		

### Hydrogen-bond geometry (Å, º)

**Table:**

*D*—H···*A*	*D*—H	H···*A*	*D*···*A*	*D*—H···*A*
O1—H1···I1^i^	0.82	3.03	3.741 (2)	146
C1—H1*B*···I3^ii^	0.97	3.05	3.924 (3)	151
C4—H4···O1^iii^	0.93	2.60	3.421 (4)	148

Symmetry codes: (i) *x*+1, *y*, *z*; (ii) *x*, −*y*+1/2, *z*−1/2; (iii) *x*, −*y*+1/2, *z*+1/2.
